# Does concomitant methotrexate confer clinical benefits in patients treated with prior biologic therapy? Analysis of data from a noninterventional study of rheumatoid arthritis patients initiating treatment with adalimumab

**DOI:** 10.1097/MD.0000000000020201

**Published:** 2020-05-08

**Authors:** Marc Schmalzing, Frank Behrens, Eva C. Schwaneck, Michaela Koehm, Gerd Greger, Holger Gnann, Harald Burkhardt, Hans-Peter Tony

**Affiliations:** a Rheumatology/Clinical Immunology, University Hospital Würzburg; b Division of Rheumatology, University Hospital Frankfurt, Goethe University; c Fraunhofer Institute for Molecular Biology and Applied Ecology IME, Project Group Translational Medicine & Pharmacology TMP, Frankfurt am Main; d AbbVie Deutschland GmbH & Co. KG, Wiesbaden; e Abteilung Biostatistik, GKM Gesellschaft für Therapieforschung mbH, München, Germany.

**Keywords:** adalimumab, biologic therapy, concomitant therapy, methotrexate, rheumatoid arthritis

## Abstract

Most studies of methotrexate (MTX) in combination with tumor necrosis factor (TNF) inhibitors have focused on treatment-naive patients with early disease. The goal of this study was to evaluate whether previous biologic therapy influenced the impact of concomitant MTX in patients initiating treatment with adalimumab.

We retrospectively analyzed data from 2 large noninterventional studies of German patients with active rheumatoid arthritis (RA) who initiated adalimumab therapy during routine clinical practice. Patients were seen between April 2004 and February 2013 for study 1 and between April 2003 and March 2013 for study 2. Key outcomes were Disease Activity Score-28 joints (DAS28), patient global assessment of health (PGA), and pain. Subgroup analyses by prior biologic treatment were performed on patients treated with continuous adalimumab monotherapy or adalimumab plus MTX for 12 months and 2-sample *t* tests were used to evaluate differences. We also assessed outcomes in subgroups in which MTX had been added or removed at 6 months and compared outcomes with 1-sample *t* tests.

Of 2654 patients, 1911 (72%) were biologic naive and 743 (28%) had received prior biologic therapy, usually with a TNF inhibitor. All subgroups showed improvements following initiation of adalimumab therapy. In patients with no previous biologic treatment, continuous adalimumab plus MTX was associated with greater improvements in DAS28, PGA, and pain at month 12 compared with continuous adalimumab monotherapy (*P* = .0006, .0031, and .0032, respectively). In patients with previous biologic treatment, concomitant MTX was associated with statistically significant benefits in pain only. Adding MTX at month 6 resulted in additional benefits in patients with no prior biologic therapy, but not those with previous biologics.

We conclude that concomitant MTX resulted in additional improvements in DAS28 and PGA vs adalimumab monotherapy in patients with no previous biologic therapy, but changes were not statistically significant in patients treated with prior biologics. These findings may help inform the patient/provider treatment decision during routine clinical care.

**KEY POINTS**

- In this retrospective analysis of patients in routine clinical practice, concomitant MTX plus adalimumab was significantly superior to adalimumab monotherapy in reducing disease activity in biologic-naive patients with RA, consistent with data from randomized clinical trials.
- However, MTX plus adalimumab did not show a significant difference to adalimumab monotherapy in reducing disease activity in RA patients previously treated with biologic therapy (primarily TNF inhibitors).
- These findings should be considered as part of the risk/benefit assessment for adding concomitant MTX in RA patients initiating therapy with adalimumab.

## 1 Introduction

The addition of methotrexate (MTX) to adalimumab, a tumor necrosis factor (TNF) inhibitor, has been shown to result in short-term and sustained long-term benefits in patients with early rheumatoid arthritis (RA), including reductions in radiographic progression.^[1–3]^ Similar observations have been made for other TNF inhibitors as well, including infliximab, etanercept, and golimumab.^[4]^ In general, however, randomized trials concerning the additional contribution of concomitant MTX compared with anti-TNF monotherapy have focused on patients with early RA.^[5]^ The effect of concomitant MTX has not been well studied in patients with long-standing disease.

In routine clinical practice, approximately one-third of RA patients treated with biologic therapy are on biologic monotherapy.^[6–9]^ However, the impact of this therapeutic choice on outcomes in the population as a whole or in specific subgroups of patients has not been systematically assessed in large-scale studies. In particular, there are minimal data available to identify subgroups of patients who may be positively or negatively affected by the decision to pursue monotherapy.

To explore the role of concomitant MTX in treatment response during routine clinical care, we used data from large noninterventional studies of patients with RA who were initiating therapy with adalimumab. Previously we reported that RA patients with prior biologic treatment experienced reduced benefit following initiation of adalimumab therapy compared with biologic naive patients, although clinically important improvements were still observed in both subgroups.^[10,11]^ The goal of this study was to evaluate whether previous biologic therapy influenced the impact of concomitant MTX in patients initiating treatment with adalimumab during routine clinical care.

## 2 Methods

### 2.1 Patients and study design

Patients in this study were enrolled in one of 2 multicenter, noninterventional cohort studies of patients treated with adalimumab during routine clinical practice in Germany that shared nearly identical study designs.^[10,11]^ Patients in the 2-year noninterventional study (NCT01077258) were seen between April 2004 and February 2013.^[10]^ Patients in the 5-year noninterventional study (NCT01078090) were seen between April 2003 and March 2013.^[11]^

To be included in the noninterventional studies, patients were required to have a diagnosis of RA, a clinical indication for treatment with a TNF inhibitor, and no contra-indications to anti-TNF therapy. All patients were informed of the study objectives and gave written consent for the anonymous use of their personal data in statistical analyses. Because of the noninterventional nature of this study, ethics approval was not required by German law. Patients in these trials were given adalimumab therapy according to routine clinical practice at the discretion of the treating clinician. The recommended dosage of adalimumab is 40 mg administered subcutaneously (SC) every other week in combination with MTX unless MTX is inappropriate.

Only patients with data recorded within 14 days of start of therapy and who received adalimumab monotherapy or adalimumab plus MTX were included in the retrospective analyses reported here. Patients who received other nonbiologic disease-modifying antirheumatic drugs (DMARDs) or biologic therapies and those with previous adalimumab therapy or inadequate data were excluded from these analyses. In addition, patients were required to have active RA (Disease Activity Score-28 joints [DAS28] ≥3.2) and information on MTX treatment at months 0, 6, and 12.

### 2.2 Assessments

Disease activity was assessed by the DAS28, a validated instrument in which higher scores indicate greater disease activity.^[12]^ The patient-reported outcomes of patient global assessment of health (PGA) and pain were assessed on 11-point categorical scales in which 0 represented the best possible status and 10 indicated the worst possible status. The analyses reported here focus primarily on assessments conducted at baseline and month 12. During the 1st year of treatment, additional assessments were conducted at months 3 and 6. All patients with available data were included in each assessment.

### 2.3 Statistical analyses

Summary statistics are presented for demographic and disease characteristics. Missing data were not imputed.

Change from baseline analyses were performed on patients with data for that outcome at baseline and month 12. Two-sided *t* tests were used to assess statistical significance. Two-sample *t* tests were used to evaluate between-group differences between the independent subgroups of adalimumab monotherapy and adalimumab plus MTX. One-sample *t* tests were used to evaluate the effect of adding or removing MTX at month 6 by assessing whether observed inter-individual differences between month 6 and month 12 were equal to 0. *P* values <.05 were considered statistically significant.

Response rates for each outcome were evaluated using previously published methods^[13,14]^ for determining critical differences (*d*_crit_) for minimum changes required for significant individual patient responses (change from baseline ≥1.8 for DAS28 and ≥3 for pain and PGA). The statistical significance of differences in response rates for patients receiving continuous adalimumab monotherapy compared with continuous concomitant MTX was assessed with 2-sided Fisher tests. Statistical analyses were performed using SAS statistical software Version 9.2 (SAS, Cary, NC).

## 3 Results

### 3.1 Baseline characteristics

Of 2654 patients included in these analyses, 1911 patients (72.0%) were biologic naive and the remaining 743 patients (28.0%) had received previous biologics. Most (710/743; 95.6%) of the patients treated with prior biologics had received at least 1 anti-TNF agent. The most common previous biologic therapies were etanercept (68%) and infliximab (40%) (patients could have more than 1 previous biologic therapy).

For subgroup analyses, patients were categorized on the basis of previous treatment with biologic therapies. Within each category (with or without previous biologics), patients were placed into subgroups on the basis of MTX therapy. Subgroups consisted of: continuous adalimumab monotherapy for 12 months; continuous adalimumab plus MTX for 12 months; addition of MTX at month 6; removal of MTX at month 6. Baseline characteristics of the subgroups were well matched for demographic characteristics (Table 1). Patients had received a mean of 2 to 3 previous DMARDs. As might be expected, patients with prior biologic therapy tended to have a longer disease duration than those with no previous biologic treatment. Most (80.5%) of the patients with prior biologic therapy had been treated with only 1 previous biologic. Approximately 3 quarters of patients were treated with systemic glucocorticoids. For patients on MTX, mean doses ranged from 11.3 to 15.6 mg/wk. Overall, patients had moderate to severe disease as indicated by DAS28 (range of 5.44–5.91 depending on the subgroup), PGA (6.14–7.10), and pain (6.38–7.24) scores.

**Table 1:**
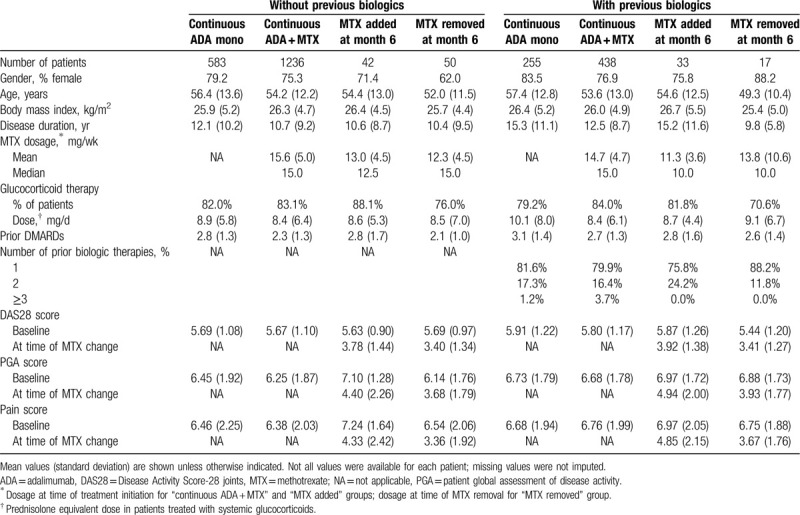
Baseline characteristics of the study population.

### 3.2 Effect of continuous concomitant MTX in patients with or without prior biologic therapy

**Figure 1:**
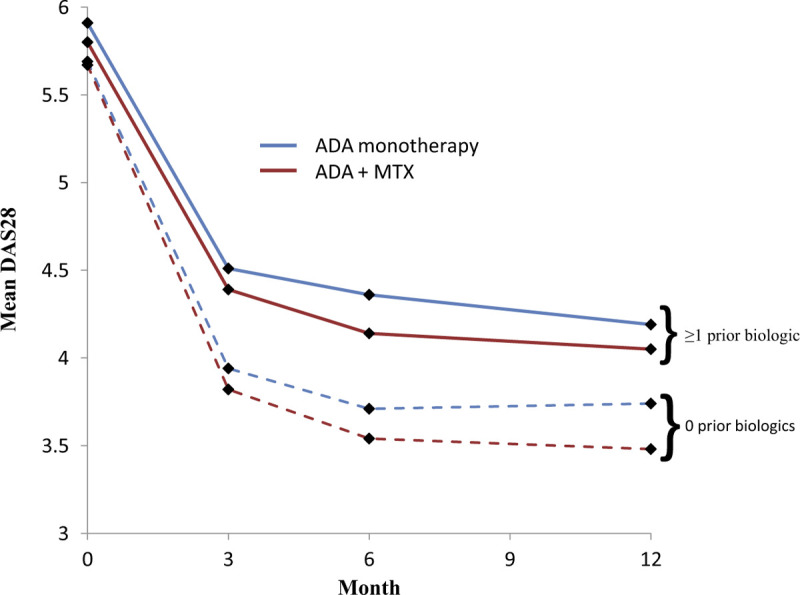
Mean DAS28 values in each subgroup during 12 months of adalimumab treatment. Blue lines = ADA monotherapy; red lines = ADA + MTX; dotted lines = no prior biologic therapy; solid lines = ≥1 prior biologic therapy. ADA = adalimumab, DAS28 = Disease Activity Score-28 joints, MTX = methotrexate.

As expected from our earlier study using all enrolled patients in the 5-year interventional study,^[11]^ all subgroups showed significant improvements in disease activity (change from baseline to month 12 in DAS28) following initiation of adalimumab therapy (*P* < .0001) (Fig. 1). A significantly improved DAS28 response rate was observed for patients with no previous biologic therapy compared with those with previous biologic therapy regardless of the use of concomitant MTX (*P* = .016 for adalimumab monotherapy and *P* < .0001 for adalimumab plus MTX; data not shown).

To explore the impact of continuous concomitant MTX on treatment response by prior biologic therapy status, we compared outcomes in patients who received continuous treatment over 12 months with adalimumab monotherapy with those who had received adalimumab plus MTX. For change from baseline analyses, patients served as their own internal control: differences in outcomes between the patient's score at baseline and their score at 12 months were calculated. The benefit of concomitant MTX was found to reach statistical significance primarily in patients with no previous biologic therapy (Table 2). Concomitant MTX resulted in significant improvements in change from baseline to month 12 and in therapeutic response rates for DAS28, PGA, and pain in patients with no previous biologic therapy compared with the changes observed in patients on adalimumab monotherapy.

**Table 2:**
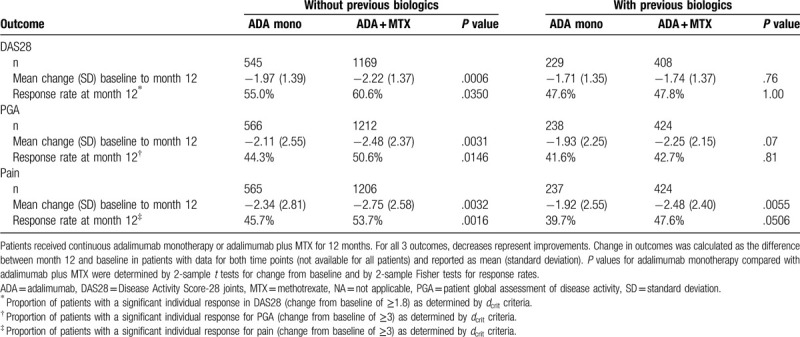
Effect of continuous concomitant methotrexate on disease activity by prior biologic therapy.

For patients with previous biologic therapy, concomitant MTX was not associated with significant improvements in DAS28 or PGA at month 12 compared with adalimumab monotherapy. However, although differences were not statistically significant, patients receiving concomitant MTX did show numerically greater reductions in disease activity (Fig. 1). For the outcome of pain in patients with previous biologic therapy, a significant improvement in change from baseline was observed in the adalimumab plus MTX subgroup compared with the adalimumab monotherapy subgroup, although the difference in therapeutic response rates for pain as calculated by *d*_crit_ criteria for significant individual changes did not achieve statistical significance (Table 2).

### 3.3 Effect of adding or removing concomitant MTX therapy during adalimumab treatment

**Figure 2:**
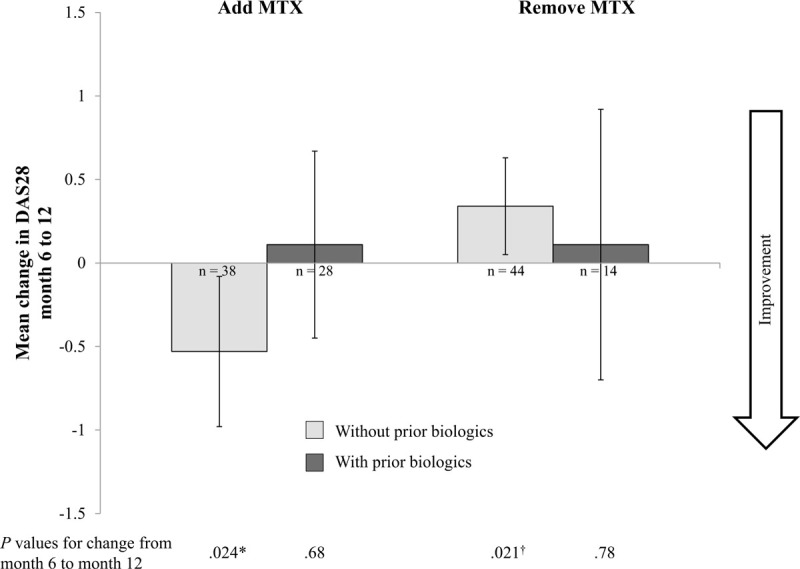
Effect of change in concomitant MTX therapy on disease activity by prior biologic therapy status. Mean change in DAS28 was calculated as the difference between month 12 and month 6 in patients with data for both time points. Capped bars indicate 95% confidence intervals. Decreases represent improvements. *P* values for change from month 6 to month 12 were determined by 1-sample *t* tests (2-sided). ADA = adalimumab, DAS28 = Disease Activity Score-28 joints, MTX = methotrexate. ∗Significant improvement in DAS28, ^†^Significant worsening in DAS28.

To explore the preferential effect of MTX further, we evaluated the impact of changes in MTX (addition or removal) at month 6 on DAS28 at month 12 (Fig. 2); each individual served as their own control. In patients without prior biologic therapy, adding MTX at month 6 resulted in a significant improvement in DAS28, and removal at month 6 resulted in a significant worsening. The addition or removal of MTX did not have a significant effect on DAS28 in patients with prior biologics. Neither PGA nor pain was significantly affected by addition or removal of MTX in any of the subgroups (data not shown).

### 3.4 Changes in glucocorticoid therapy in patients receiving continuous concomitant MTX

The favorable effect associated with MTX in patients without previous biologic therapy could potentially be explained by a therapeutic response mediated by increased use of systemic glucocorticoid therapy in the biologic-naive subgroup receiving concomitant MTX. Although the proportions of patients receiving systemic glucocorticoids at baseline were comparable for patients receiving continuous concomitant MTX with or without previous biologic therapy (Table 1), by month 12 the proportion of patients receiving glucocorticoids in the adalimumab plus MTX subgroup with no previous biologic treatment was markedly reduced (65.6%) compared with the adalimumab plus MTX subgroup treated with prior biologic therapy (75.3%), and the mean dose was similarly reduced (from 8.4 mg/d at baseline in both groups to 5.1 mg/d in patients on adalimumab plus MTX without prior biologics and 5.8 mg/d in those with prior biologics). These findings are consistent with an improved therapeutic response in the adalimumab plus MTX subgroup without prior biologic therapy. We therefore conclude that a greater use of systemic corticosteroids does not account for the improvements observed with MTX therapy in biologic-naive patients.

## 4 Discussion

The availability of a large cohort of RA patients initiating treatment with adalimumab provided the opportunity to explore the effect of concomitant MTX therapy in patients with or without prior biologic therapy. In this study, we found that RA patients with no previous biologic therapy benefited from the combination of MTX and adalimumab compared with adalimumab alone. This was observed both for DAS28 and for the patient-reported outcomes of PGA and pain. In contrast, patients with prior biologic therapies benefited from treatment with adalimumab, but the addition of concomitant MTX did not result in significant additional improvements in DAS28 or PGA compared with adalimumab monotherapy. For the outcome of pain, patients with prior biologic therapy did show a significantly greater change from baseline to month 12 with concomitant MTX, but no difference in the rate of individual responses compared with monotherapy.

To further test the hypothesis that MTX was associated with benefit in patients with no prior biologics compared with those receiving previous biologics, we evaluated month 12 outcomes in subgroups of patients who added or stopped MTX at month 6. Patients served as their own controls in these analyses, thus eliminating confounding factors associated with analyses of population means. Although sample sizes were small, the subgroup analyses supported the earlier conclusion that concomitant MTX provides greater benefits in biologic-naive patients than in those who have been treated with prior biologics.

A large body of evidence supports the beneficial effects of combination therapy with TNF inhibitors and MTX compared with biologic monotherapy alone, including the adalimumab PREMIER trial.^[1]^ In the PREMIER trial, combination therapy with adalimumab plus oral MTX (20 mg/wk) was superior to adalimumab alone and to MTX alone at 2 years,^[1]^ and the benefits of combination therapy extended up to 10 years.^[2]^ However, the PREMIER trial only enrolled MTX-naive patients with early RA (<3 years duration).^[1]^ The patient population analyzed in the PREMIER trial was thus quite different from the population described here, which had a mean disease duration of 10 to 15 years and had been treated with a mean of 2 to 3 previous DMARDs (depending on the subgroup).

The appropriate usage and optimization of MTX in patients with RA is still evolving. There is evidence that parenteral administration, including SC MTX, may be more effective than oral therapy, especially at higher doses.^[15,16]^ Current European League Against Rheumatism guidelines for RA recommend a rapid escalation of MTX to a dose of 25 to 30 mg/wk for MTX monotherapy, but do not provide dosing guidelines for MTX in combination with TNF inhibitors.^[17]^ As mentioned previously, the adalimumab PREMIER trial used an oral MTX dose of 20 mg/wk.^[1]^ In the CONCERTO trial in patients with early biologic and MTX-naive RA receiving treatment with adalimumab, patients receiving concomitant therapy with oral MTX 10 mg/wk had almost identical outcomes to those receiving therapy with oral MTX 20 mg/wk.^[18]^ However, in patients with established RA receiving oral MTX at ≥15 mg/wk for at least 12 weeks before initiating adalimumab, patients who were randomized to low dosage (7.5 mg/wk) vs high dosage (20 mg/wk) MTX in combination with adalimumab had slightly less favorable outcomes, although differences were minor.^[19]^ The best dosage of MTX in combination with adalimumab thus remains unclear, although oral doses between 10 and 20 mg/wk appear to achieve largely the same results. In our study, patients received MTX at 11.3 to 15.6 mg/wk, depending on the subgroup. Although it is possible that some of the patients on MTX in our study were suboptimally dosed, based on previous studies it seems likely most patients were receiving adequate doses of MTX to confer a clinical benefit.

The diminished effect of concomitant MTX in patients with previous biologic treatment could potentially be due to several different or overlapping factors, including (but not limited to) reduced responses to MTX in patients with longer disease durations or other patient characteristics specific to the subgroup treated with prior biologics, curtailed responses in patients with any form of previous treatment, reduced MTX effects on antidrug antibody formation in patients receiving prior biologics, or alterations in the inflammatory course of the disease during prior biologic therapy that decreases the impact of MTX. There is some evidence that previous nonbiologic DMARD therapy is associated with a reduced response to MTX in patients receiving MTX monotherapy^[20]^ or MTX in combination with biologic therapy,^[5]^ suggesting that patients who receive any previous RA treatment may be more refractory to the therapeutic effects of MTX than treatment-naive patients. It is therefore possible that in the background of the overall lower response to TNF inhibitors in patients treated with previous biologics,^[11,21]^ the incremental benefit of concomitant MTX decreases to the level that a statistically significant difference can no longer be detected. This explanation is supported by the consistent but nonsignificant difference in response observed with concomitant MTX compared with adalimumab monotherapy in patients treated with prior biologics at all time points during the 1st year after initiation of adalimumab therapy.

Another possible explanation for our observations is a reduced effect of MTX on antidrug antibody formation in patients who have received previous biologic therapy compared with biologic-naive patients. MTX is known to reduce the immunogenicity of adalimumab,^[22]^ and concomitant MTX is associated with improved drug survival for TNF inhibitors.^[23,24]^ It is possible that this effect is modified by previous biologic therapy. Alternatively, previous treatment with biologics may alter the inflammatory course of the disease to an extent that the benefits of MTX are less pronounced. The significant effect of MTX on self-reported pain in patients regardless of previous biologic treatment may indicate that pathways associated with pain are additional to or different from those that moderate disease activity. Further studies will be required to explore these possibilities for the differential effect of MTX by prior biologic therapy.

The relevance of a better understanding of the impact of concomitant MTX on clinical outcomes is highlighted by the high proportion of patients on biologic monotherapy in routine clinical practice. For some patients, the benefits of concomitant MTX may be outweighed by tolerability issues,^[25]^ which are potentially exacerbated by a low patient awareness of issues relevant to MTX safety.^[26]^ In this observational study, 32% of patients received adalimumab monotherapy; this figure is consistent with the 25% to 40% of RA patients reported to receive biologic monotherapy during routine clinical care.^[6–9]^ These numbers may underestimate the use of biologic monotherapy. A recent study found that clinicians misclassify MTX use in up to 20% of patients, usually because the patient has discontinued MTX therapy or missed doses.^[27]^ It is of interest that 2 studies have reported that biologic monotherapy is more common in patients with previous biologic therapy than in biologic-naive patients.^[6,8]^ The reduced use of MTX with previous biologic therapy could be due to tolerability issues in more heavily treated patients,^[25]^ or could potentially reflect clinical experience with reduced response in patients with prior biologic therapy treated with concomitant MTX, as we have found in the study reported here.

### 4.1 Limitations

The study reported here was not randomized, and the treatment decision of whether to add concomitant MTX was likely influenced by patient characteristics as well as physician and patient preferences, thereby reflecting real-world clinical care. As with all retrospective observational studies, potentially confounding factors, including patient characteristics and varying MTX doses, could influence outcomes and provide alternate explanations for the differences observed here. Missing data may also have influenced our findings. Accordingly, the data from this observational study support an interesting hypothesis concerning reduced MTX activity in patients treated with prior biologics, but randomized trials will be required to confirm this finding. In addition, the conclusions from this study are restricted to combination therapy with adalimumab plus MTX and are primarily confined to patients receiving previous biologic therapy with TNF inhibitors, as more than 95% of the prior biologic patients in this study had received treatment with at least 1 TNF inhibitor. Further studies will be needed to determine whether these observations apply to other biologic therapies, including those with a different mechanism of action. The reduced benefit observed with TNF inhibitors in patients treated with prior biologic therapy is a common finding not restricted to adalimumab.^[21]^ We therefore consider it likely that our results concerning adalimumab and concomitant MTX in patients with prior biologic therapy can be extrapolated to other TNF inhibitors as well. However, additional studies will be required to confirm this hypothesis.

## 5 Conclusion

In conclusion, our study provides strong support for continuous concomitant MTX therapy in patients initiating adalimumab who have not received previous biologic therapy. Although initial administration of combination therapy is optimal, the addition of MTX at a later time point results in statistically significant improvements in outcomes in biologic-naive patients. On the contrary, if these patients stop MTX comedication, it is important to be aware that they may lose some disease control. For patients treated with prior biologic therapy in this study, concomitant MTX did not result in significant improvements in the effectiveness of adalimumab with respect to DAS28 or PGA, but there were some benefits on pain and modest improvement in other parameters that may have had clinical significance to individual patients. The findings from this study should be used to help inform the patient/provider decision on the use of adalimumab monotherapy vs adalimumab plus MTX in the treatment of RA.

## Acknowledgment

The authors thank Sharon L. Cross, PhD, who provided medical writing services on behalf of CIRI, Frankfurt am Main, Germany, under contract with AbbVie Deutschland GmbH & Co. KG for medical writing services.

## Author contributions

**Conceptualization:** Marc Schmalzing, Frank Behrens, Eva C. Schwaneck, Michaela Koehm, Gerd Greger, Harald Burkhardt, Hans-Peter Tony.

**Data curation:** Holger Gnann.

**Formal analysis:** Gerd Greger, Holger Gnann.

**Investigation:** Marc Schmalzing, Frank Behrens, Eva C. Schwaneck, Michaela Koehm, Gerd Greger, Holger Gnann, Harald Burkhardt, Hans-Peter Tony.

**Methodology:** Marc Schmalzing, Frank Behrens, Gerd Greger, Holger Gnann, Harald Burkhardt, Hans-Peter Tony.

**Project administration:** Gerd Greger.

**Writing – original draft:** Marc Schmalzing, Hans-Peter Tony.

**Writing – review & editing:** Marc Schmalzing, Frank Behrens, Eva C. Schwaneck, Michaela Koehm, Gerd Greger, Holger Gnann, Harald Burkhardt, Hans-Peter Tony.
